# Editorial

**Published:** 1874-06

**Authors:** 


### Editorial.

***Who is He?***

We mean the Chicago physician who is being advertised extensively in the rural medical journals as having been presented with a silver mounted skeleton by his admiring friends. We fear some of our bucolic friends have been hoaxed.

The *Philadelphia Medical and Surgical Reporter* tells a new (!) story of a Chicago physician, also a real estate operator, directing his patient to take her medicine "a quarter down, and the balance in one, two and three years." This was a very good story about thirty years ago, *i. e.*, about twenty years before the alleged author was himself invested in real estate "dust to dust."

"The cry is, Still they come." *The Medical News*, copied by the *Times* and *Clinic*, enumerates the graduates of the various medical schools, omitting entirely the seventy-seven who claim Rush Medical College as their *Alma Mater.* If the lists are not already filled, will the journals referred to be kind enough to make a little room for these, as some of them are really deserving of mention, numerically, at least.

***Directory for the Month.***

We take the liberty to call the attention of those of our readers most directly interested, to the "Directory for the Month," to be found on the last page of each number of the JOURNAL. It was designed for the especial benefit of members of the profession visiting the city (or residents) who may desire to know where a leisure hour may be passed both pleasantly and profitably.

An examination of this page of the JOURNAL will show that very few hours in the week are unimproved by the large corps of professional teachers and pupils to be found in this city. It will show, moreover, that the "facilities for acquiring a medical education" are quite as great in Chicago as can be utilized advantageously by the most diligent student, who will have no one to blame but himself for every idle hour. We trust that those interested will notify us of any corrections necessary to make the directory accurate and complete. H.

***The Dignity of the Profession—***

Might be the text, were one disposed to be sarcastic: the commentary thereupon—a sharper than which the keenest sarcasm would fail to write—would be the recent scramble for the office of City Physician, so lately terminated in the Common Council of Chicago, and the remarks of the daily press upon contest and contestants. How is it possible for the medical profession as a body to expect deference and respect from the public, when its members condescend year after year to take part in these undignified struggles for paltry municipal offices? In that which has just terminated there were ten or a dozen participants, whose names, professional reputations and private characters were discussed in the Council and in the public press in the manner and style of pot-house politicians. If the one-half of the derogatory remarks made of them was true, the large majority was totally unfit for the office of City Physician, or, indeed, for any other office of trust or emolument whatever; if, on the other hand, the comments were untrue, then surely no respectable physician should ever consent to place himself in a position in which his personal and professional pride must sustain such bitter humiliation.

Some, unfamiliar with the facts, might ask, For what great prize is this contest sustained? Is the position one of such dignity and honor, or so well paid pecuniarily, as to compensate for the odium of the contest for its possession? The dignity and honor is all that which can be gleaned in the cells of the city prisons and station houses, and the compensation a little larger than that which is paid to the veterinary surgeon for caring for the sick horses of the fire department.

The members of the medical profession would much better advance their own interest, individually and collectively, by keeping aloof from such unseemly contests until the office should seek the man, not the man the office. They could then dictate the terms and conditions of acceptance, and thus become masters of the situation, and not, as now, the mere appendages of some petty ward politician. Members of the profession, appreciative of the demoralizing influences to which the holders of these petty municipal offices are constantly subjected, are never found amongst the applicants for such positions; for it is certain that few who allow themselves to be thus drawn through the mire of municipal politics escape without a stain. If young men would perceive the fact that the occupancy of such positions has hitherto been an obstacle rather than an aid to professional advancement, they would avoid rather than seek them. Local administrations needing medical services would be compelled to seek them, and moreover to pay adequately for them when found. H.

***The Medico-Historical Society.***

Two years ago two physicians of this city issued a Medical Register and Directory, containing "a description of the medical colleges, hospitals, infirmaries, asylums and charitable institutions, together with the medical and other scientific associations of the State of Illinois."

This was subjected to the revision of "the presidents of the regular medical colleges and societies of Chicago, and of the Illinois State Medical Society."

This enterprise was an effort in the right direction, and although by no means perfect, received, as it merited, general commendation. But this commendation was not universal, and the projectors of the Register were violently assailed, and threatened with vengeance dire by some whose names were excluded from the published list of reputable practitioners.

In order to avoid the personal annoyance to which individuals could not fail to be subjected in the publication of such a work, it was deemed advisable to organize a society which should comprise this amongst certain other duties. With this end in view, a call was issued to a large number of physicians to meet for the purpose of effecting such an organization.

Thus far all seemed well; the intention was commendable, and the mode of its execution apparently correct, but there was in that call an error, the first of several since committed, which may prove radical obstacles to the growth and efficiency of a well-intended institution.

Error number one consisted in the calling of the meeting at the office of a physician instead of in a public hall. The movement should have been exempt from the slightest suspicion that it originated under the auspices of any one man or party. Such suspicion could not fail to arise in the minds of many, under the circumstances surrounding this meeting, who would thereby (unwisely, indeed,) withhold from it their sanction or co-operation.

To obviate the consequences of this error, an effort was made, by one who comprehended the situation, to remove the consideration of the whole subject to another time and place, when and where it should be presented to the profession under the auspices of a committee selected for that purpose.

The second error was involved in the organization of this original meeting, by the selection for its officers of gentlemen (unexceptionable otherwise) holding similar positions in a medical society already in active operation, thereby suggesting the relation of subordination of the proposed to the society above referred to.

Error number three was made in the appointment of the committee of organization, which should not have been selected exclusively from those in attendance upon that meeting, about one-tenth of the numerical strength of the profession, but should have been more generally representative in its constitution.

Error number four, and in our humble judgment the most serious of all, both in principle and in practice, was the organization of the society by wholesale, those in attendance at the meeting resolving themselves into a society, etc., etc., etc., and this coincidently with the adoption of a constitution and by-laws providing definitely for the election of members by ballot, etc., etc. Herein was violated one of the radical principles of their organization, the practical effect of which may be to encumber the society with useless or objectionable material, of which it will be powerless to rid itself hereafter.

These remarks are designed to reflect invidiously upon no one identified with the movement, but are made under a strong conviction of the necessity for such an organization, of the good faith of its projectors, and at the same time, however, with a tolerably comprehensive acquaintance with the history of the profession in the city during the last seventeen years, and an accurate appreciation of its present status. In truth, we are not altogether a "happy family," that is, not yet—although we hope to be some day. We have skeletons in our closet—the skeletons of envy, hatred and malice—and should use every effort to keep them locked up until they moulder into dust, and not allow them to escape and mingle in medico-historical or any other organizations. "Eternal vigilance is the price of" peace. H.

For the CHICAGO MEDICAL JOURNAL.

In the JOURNAL for May, "A Country Correspondent" indulges in a criticism of the valedictory address which was printed in the April No. That the address should elicit criticism is not surprising, for subjects are involved upon which the best intellects of the world are discordant. That some of the exceptions are not well taken will appear by a few citations.

The address should *not* "have commenced with the first paragraph on page 195," but with the first paragraph on page 193, for in this paragraph a simple fact is stated which was intended to be, and is applied in illustration in the last paragraph commencing on page 201. The analogy between the physical world and the intellectual, as sketched in the second paragraph on page 193, appears natural, and it is maintained throughout the address.

The reference to the allegory in the first paragraph on page 194, was introduced there, that the lesson which it was intended to teach might be applied with effect in the first paragraph on page 206. (All of which is legitimately a part of the address.)

It is asserted that the quotation from the Bible, on page 201, is introduced without "pausing to study patiently in the light of modern science." The real object in this part of the address was to bring the teachings of scientists and revelation into contact.

Time, as an element, modifying results in nature's laboratory, and applicable to this part of the subject, is recognized in the second paragraph on page 200, and again in the first paragraph on page 201.

The address should *not* end on page 203, as the reviewer intimates, though "enough had been said to arouse the attention of the graduating class," for then should be added some words to stimulate to, and encourage in, *personal* efforts in the work of progress.

Exception is taken to the use of certain terms, as, *e. g.*, "disease," "poison," "cure." The reviewer should know that these terms are used to-day by the scientific, and also that they convey definite meaning. In the distant future when improvement shall have furnished us with better terms to express the idea, they shall be marshalled into service.

In conclusion, it is hoped that the praise, so liberally awarded to the address, has better foundation than these points in the critique.

DE LASKIE MILLER.

926 Wabash Av.

---

### *Chicago Mortality Report for April, 1874.*

MORTALITY IN MONTH OF APRIL, 1874.

| | |
|---|---|
| Accident, by burns | 3 |
| " by being crushed | 2 |
| " by drowning | 5 |
| " by fall | 1 |
| " by railroad | 5 |
| " by scalding | 3 |
| " by street cars | 1 |
| Apoplexy | 2 |
| Asthma | 1 |
| Atelectasis pul | 1 |
| Brain, congestion of | 3 |
| " disease of | 3 |
| " inflammation of | 7 |
| " softening of | 3 |
| Bronchitis | 8 |
| " capillary | 4 |
| Cancer of liver | 1 |
| " of stomach | 2 |
| " of uterus | 1 |
| Carbuncle | 1 |
| Chicken Pox | 1 |
| Cholera Infantum | 1 |
| Consumption | 54 |
| Convulsions | 58 |
| Croup | 1 |
| Cyanosis | 3 |
| Cystitis | 1 |
| Debility, general | 6 |
| Deficient development | 1 |
| Diarrhœa | 4 |
| Diphtheria | 7 |
| Dropsy, general | 6 |

| | |
|---|---|
| Dysentery | 4 |
| Enteritis | 22 |
| Epilepsy | 4 |
| Erysipelas | 4 |
| Fever, congestive | 2 |
| " intermittent | 1 |
| " puerperal | 11 |
| " remittent | 4 |
| " scarlet | 5 |
| " typhoid | 11 |
| Gastritis | 3 |
| Gastro Enteritis | 3 |
| Hemorrhage, puerperal | 1 |
| Heart, disease of | 4 |
| " organic disease of | 1 |
| " rheumatism of | 1 |
| " inflammation of | 1 |
| " valvular disease of | 9 |
| Hip disease | 1 |
| Hemiplegia | 2 |
| Hernia, umbilical | 1 |
| Hepatitis | 2 |
| Hydrophobia | 1 |
| Hydrocephalus | 8 |
| Inanition | 10 |
| Kidneys, Bright's disease of | 3 |
| Laryngitis | 2 |
| Larynismus, stridulous | 1 |
| Larynx, inflammation of | 1 |
| Leucocythemia | 1 |
| Lungs, abscess of | 1 |
| " congestion of | 10 |
| " hemorrhage of | 1 |
| Malformation | 1 |
| Mortification, following amputation | 1 |
| Meningitis | 10 |
| " cerebro spinal | 7 |
| " tubercular | 2 |
| Metro Peritonitis | 1 |
| Old Age | 12 |
| Paralysis | 3 |
| Parotitis | 1 |
| Peritonitis | 1 |
| Pneumonia | 44 |
| " typhoid | 4 |
| Pleurisy | 1 |
| Pyæmia | 1 |
| Rheumatism | 1 |
| " inflammatory | 1 |
| Septicæmia | 1 |
| Small-Pox | 17 |
| Spine, disease of | 1 |
| Stomach, ulceration of | 1 |
| Suicide, cutting throat | 1 |
| " hanging | 1 |
| " poison | 2 |
| " strychnine | 2 |
| " shooting | 3 |
| Syphilis | 1 |
| Tabes Mesenterica | 10 |
| Teething | 3 |
| Tetanus | 2 |
| Trismus | 1 |
| Uterus, hemorrhage of | 1 |
| " degeneration of | 1 |
| Whooping Cough | 7 |
| Total | 482 |

Premature births, 5 ; Still births, 63. Total ........ 68

COMPARISON.

| | | |
|---|---|---|
| Deaths in month of April, 1874 | | 482 |
| " " March, 1874 | | 508 |
| Decrease | 26 | |
| Deaths in month of April, 1873 | | 611 |
| Decrease | 129 | |

AGES.

| | |
|---|---|
| Under one year | 144 |
| One year to two | 39 |
| Two years to three | 16 |
| Three " " four | 14 |
| Four " " five | 14 |
| Five " " ten | 27 |
| Ten " " twenty | 13 |
| Twenty " " thirty | 50 |
| Thirty " " forty | 47 |
| Forty years to fifty | 40 |
| Fifty " " sixty | 28 |
| Sixty " " seventy | 26 |
| Seventy " " eighty | 18 |
| Eighty " " ninety | 5 |
| Ninety " " one hundred | 1 |
| Total | 482 |

| | |
|---|---|
| Colored | 11 |
| White | 471 |
| Total | 482 |
| Males | 245 |
| Females | 237 |
| Total | 482 |

Married, 172 ; Single, 310. Total ........ 482

NATIVITIES.

| | | | |
|---|---|---|---|
| Austria | 2 | Holland | 1 |
| Bohemia | 3 | Italy | 1 |
| Canada | 1 | Ireland | 46 |
| Native—Chicago | 63 | Norway | 6 |
| Foreign, " | 151 | Poland | 3 |
| United States, other parts | 97 | Scotland | 1 |
| Denmark | 1 | Sweden | 6 |
| England | 15 | Switzerland | 4 |
| France | 2 | Unknown | 4 |
| Germany | 75 | Total | 482 |

Deaths daily, 16. Mean thermometer, 38.7°. Rain fall, 2.64 inches.

MORTALITY BY WARDS, ETC., APRIL, 1874.

| Wards. | No. Deaths. | Pop. in 1872. | Percentage. |
|---|---|---|---|
| 1 | 4 | 398 | one death in ---- |
| 2 | 3 | 2,174 | " " " ---- |
| 3 | 33 | 19,157 | " " " 581 |
| 4 | 10 | 16,832 | " " " 1,683 |
| 5 | 17 | 18,564 | " " " 1,092 |
| 6 | 53 | 31,371 | " " " 592 |
| 7 | 43 | 27,644 | " " " 643 |
| 8 | 21 | 30,253 | " " " 1,441 |
| 9 | 24 | 30,032 | " " " 1,249 |
| 10 | 13 | 16,624 | " " " 1,278 |
| 11 | 11 | 18,340 | " " " 1,667 |
| 12 | 15 | 20 876 | " " " 1,392 |
| 13 | 13 | 14,636 | " " " 1,126 |
| 14 | 13 | 15,892 | " " " 1,222 |
| 15 | 35 | 40,047 | " " " 1,144 |
| 16 | 35 | 19,099 | " " " 543 |
| 17 | 22 | 17,513 | " " " 796 |
| 18 | 30 | 19,977 | " " " 666 |
| 19 | 3 | 2,944 | " " " ---- |
| 20 | 7 | 5,023 | " " " ---- |
| | 405 | Ratio of deaths to population in 1872, one death in 721. | |

| | | | |
|---|---|---|---|
| No. deaths in Wards | 405 | Mercy Hospital | 4 |
| Accidents | 20 | Police Station | 1 |
| Alexian Brothers Hospital | 1 | Small Pox Hospital | 1 |
| County Hospital | 16 | St. Luke's Hospital | 2 |
| Foundlings' Home | 18 | St. Joseph's " | 2 |
| Hahneman Hospital | 1 | Suicides | 9 |
| Home of Friendless | 2 | Total | 482 |

CASES OF SMALL-POX REPORTED DURING APRIL, 1874.

| Wards. | No. Cases. | Wards. | No. Cases. | Wards. | No. Cases. |
|---|---|---|---|---|---|
| 1 | 1 | 8 | 3 | 15 | 11 |
| 2 | -- | 9 | 4 | 16 | 9 |
| 3 | -- | 10 | 1 | 17 | 15 |
| 4 | -- | 11 | 4 | 18 | 6 |
| 5 | -- | 12 | -- | 19 | -- |
| 6 | 5 | 13 | -- | 20 | -- |
| 7 | 4 | 14 | -- | | |

Total 63

Cases reported during April, 1874 63
" " " March, 1874 45
Increase 18
Cases reported during April, 1873 107
Decrease 107

***Directory for the Month.***

*Every Monday. Lectures.*—At Rush College, 9 to 1 o'clock.—Drs. Wadsworth, Bridge, Jackson and Parkes. At the Chicago College, 8 to 12 o'clock. Drs. Quine, Curtis and Roler. At Woman's Hospital College, 9 to 11 o'clock. Drs. Dyas and Fitch.

*Clinics.*—At Mercy Hospital, 3 P. M. Surgery.—Dr. Andrews. At the Central Dispensary, (239 W. Van Buren street), 3 P. M. Medical.—Dr. Bridge. At the Eye and Ear Infirmary, 2 P. M.—Dr. Holmes. At Hospital for Women and Children, 1.30 P. M. Gynæcological.—Dr. Thompson.

*Mondays, May 4th and 18th.*—Meeting of *Chicago Medical Society*, in the evening, at Gault House.

*Mondays, May 11th and 25th.*—Meeting *Chicago Association of Physicians and Surgeons*, in the evening, at Grand Pacific Hotel.

*Every Tuesday. Lectures.*—At Rush College, 9 to 1 o'clock.—Drs. Owens, Bridge, Adolphus and Parkes. At 4 P. M.—Dr. I. N. Danforth. At Chicago College, 8 to 12 o'clock.—Drs. Bond, Earle, Hutchinson and F. H. Davis. At Woman's Hospital College, 8 to 10 A. M.—Drs. McDonald and Bartlett.

*Clinics.*—At County Hospital, 2 P. M. Surgical.—Dr. Bogue. Medical.—Dr. Bevan. At Mercy Hospital, 3 P. M. Gynæcological.—Dr. Roler.

*Tuesday, 19th inst.—Illinois State Medical Society.* (Place announced by daily prints.)

*Tuesday, 12th inst.*—7.30 P. M., at 263 Wabash avenue, *Chicago Academy of Sciences.*

*Every Wednesday. Lectures.*—At Rush College, 9 to 1 o'clock.—Drs. Wadsworth, E. F. Ingals, Strong and Parkes. At Chicago College, 8 to 12 o'clock.—Drs. Stilliaus, Sherman, Nelson and Merriman. At Woman's Hospital College, 9 to 11 A. M.—Drs. Thompson and Fisher.

*Clinics.*—At County Hospital, 2 P. M. Gynæcological.—Dr. Quine, 3 P. M. Ophthalmological.—Dr. Hotz. At Mercy Hospital, 3 P. M. Ophthalmological.—Dr. S. J. Jones. At St. Luke's Hospital, 8 A. M. Surgical.—Dr. Owens. At Central Dispensary, 3 P. M.—Dr. Bridge.

*Every Thursday. Lectures.*—At Rush College, 9 to 1 o'clock.—Drs. Hyde, Bridge, Jackson and Case. At Chicago College, 8 to 12 o'clock.—Drs. Quine, Earle, Hutchinson and Haines. At Woman's Hospital College, 3 to 6 P. M. Drs. Paoli, Blake and Delafontaine.

*Clinics.*—At Mercy Hospital, 3 P. M. Medical.—Dr. Merriman. At Central Dispensary, 2 P. M. Gynæcological.—Dr. Adolphus. Diseases of Chest. Dr. E. F. Ingals. At Hospital for Women and Children, 1.30 P. M. Medical.—Dr. A. H. Foster.

*Every Friday. Lectures*—At Rush College, 9 to 1 o'clock.—Drs. Wadsworth, Hayes, Adolphus and Case. At 4 P. M.—Dr. Danforth.

*Clinics.*—At County Hospital, 2 P. M. Medical.—Dr. Bevan. Surgical—Dr. Bogue. At Mercy Hospital, 3 P. M. Medical.—Dr. Nelson.

*Every Saturday. Lectures.*—At Rush College, 9 to 12 o'clock.—Drs. Owens, Hay and Strong. At Chicago College, 8 to 12 o'clock.—Drs. Bond, Jewell and Haines At Woman's Hospital College, 10 to 12 o'clock.—Drs. Curtis and McDonald.

*Clinics.*—At Rush College, 2 P. M. Surgery.—Dr. Gunn. 3 P. M. Diseases of Nervous System.—Dr. Hay.

